# Supplementary material for: The binding mechanism of an anti-multiple myeloma antibody to the human GPRC5D homodimer
Source: Nat Commun. 2024 Jun 19;15:5255. doi: 10.1038/s41467-024-49625-y (PMC11187071; doi:10.1038/s41467-024-49625-y)
Supplement: Supplementary file 1 — Supplementary Information [file 41467_2024_49625_MOESM1_ESM.pdf]

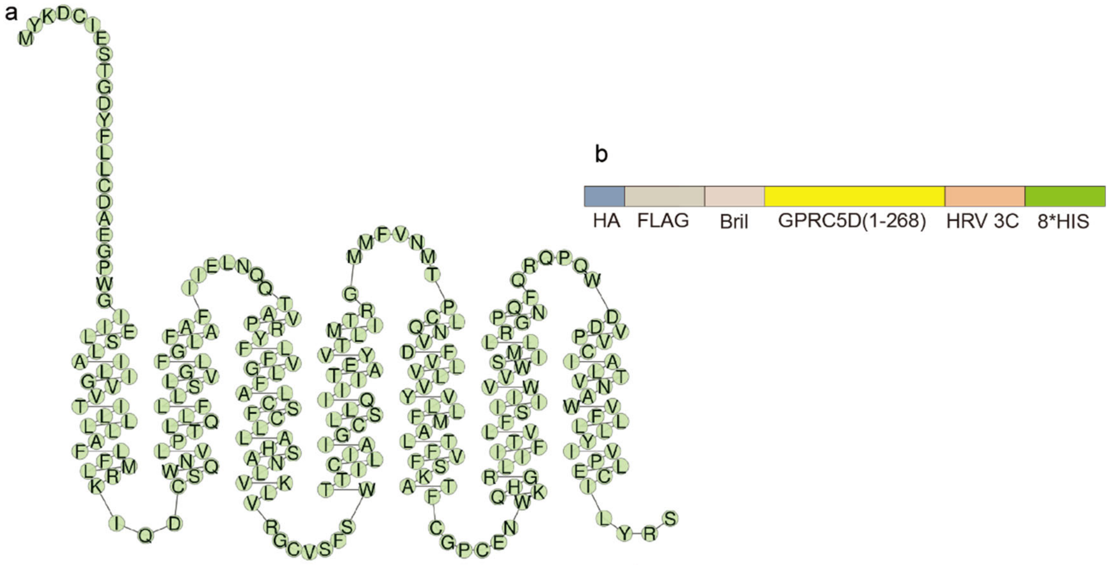

c Consensus analysis of Class C GPCRs (7TM region)

Receptors	Identity %	Similarity %
GPRC5D	100	100
GPRC5A	55	73
GPRC5B	41	65
GPRC5C	44	63
mGluR$_3$	22	47
CaSR	23	42
GPRC6A	21	41
mGluR$_2$	19	41
mGluR$_4$	17	41
mGluR$_1$	19	41
mGluR$_7$	19	41
mGluR$_8$	17	40
mGluR$_5$	20	40
mGluR$_6$	19	39
TAS1R$_1$	15	37
GABA$_{B1}$	17	36
GABA$_{B2}$	13	35
TAS1R$_2$	17	34
TAS1R$_3$	16	30
GPR156	10	30
GPR158	10	29
GPR179	12	29

d Consensus analysis of Class C GPCRs (Full length)

Receptors	Identity %	Similarity %
GPRC5D	100	100
GPRC5A	37	51
GPRC5B	25	42
GPRC5C	25	38
mGluR$_3$	5	12
CaSR	5	12
GPRC6A	5	12
mGluR$_2$	5	12
mGluR$_4$	5	12
mGluR$_1$	5	11
mGluR$_7$	5	11
mGluR$_8$	4	11
mGluR$_5$	4	11
mGluR$_6$	5	10
TAS1R$_1$	4	10
GABA$_{B1}$	4	10
GABA$_{B2}$	4	9
TAS1R$_2$	4	9
TAS1R$_3$	4	9
GPR156	4	8
GPR158	3	7
GPR179	2	4

Supplementary Fig 1 | GPRC5D construct for cryo-EM study. a Snake plot of hGPRC5D (1-268). **b** The illustration of construct used for cryo-EM study. **c**, **d** The sequence identity and similarity for both the 7 transmembrane helical (7TM) region (**c**) and full-length receptor (**d**) of all Class C GPCRs are generated from the "Receptor Similarity" function in GPCRDB database. The red box highlights the GPRC5 subfamily.

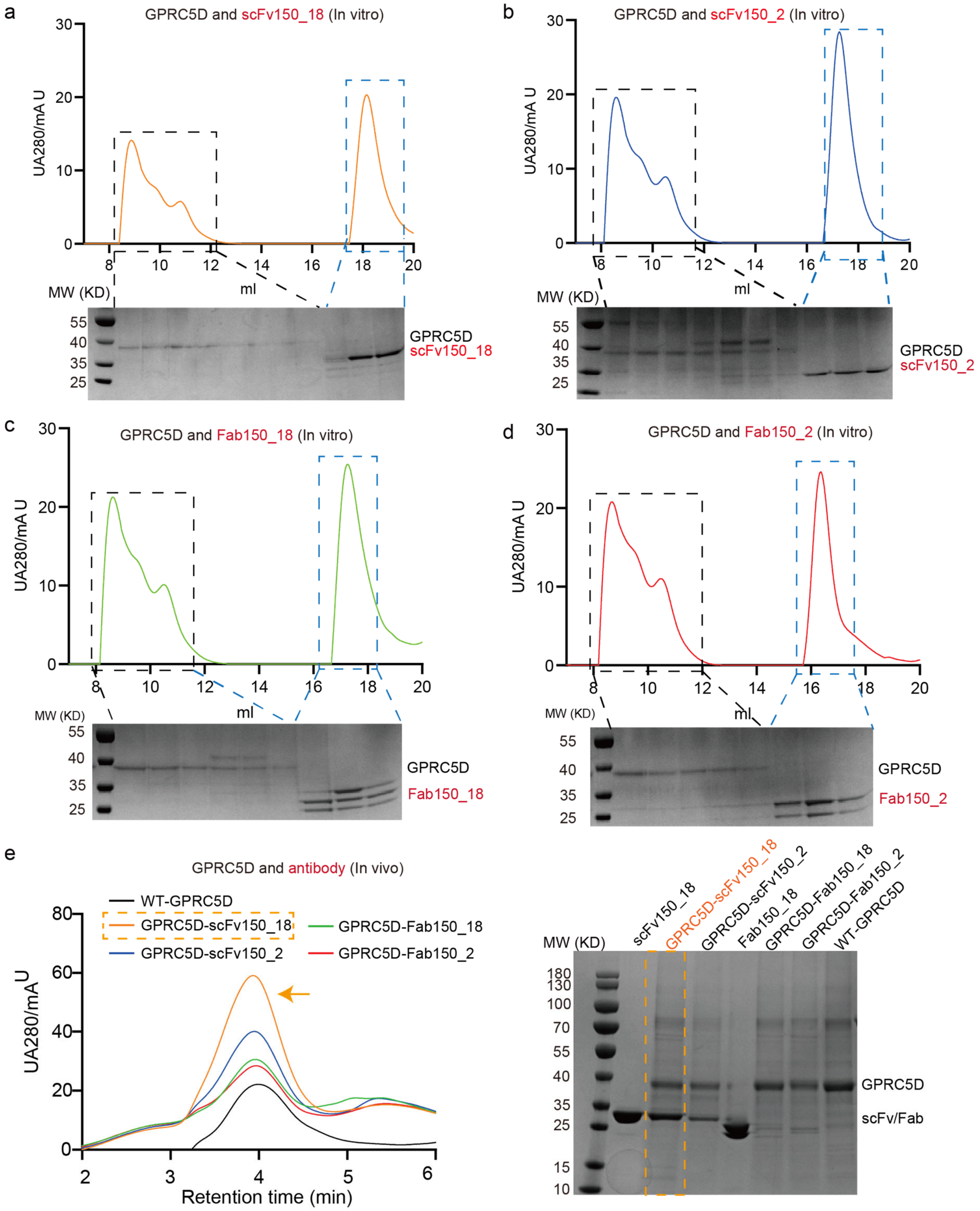

Supplementary Fig 2 | Screening of GPRC5D antibodies for complex formation. a-d The in vitro assembly results of four antibodies with GPRC5D. Size-exclusion chromatography (SEC, top) and SDS-PAGE gels (bottom) of the GPRC5D-antibody complexes. **e** The results of co-expression of four antibodies with GPRC5D in insect cells. Left panel, analytical SEC of the GPRC5D-antibody complex, and, right panel, SDS–PAGE analysis of the complex sample. The best one has been marked in orange dashed box.

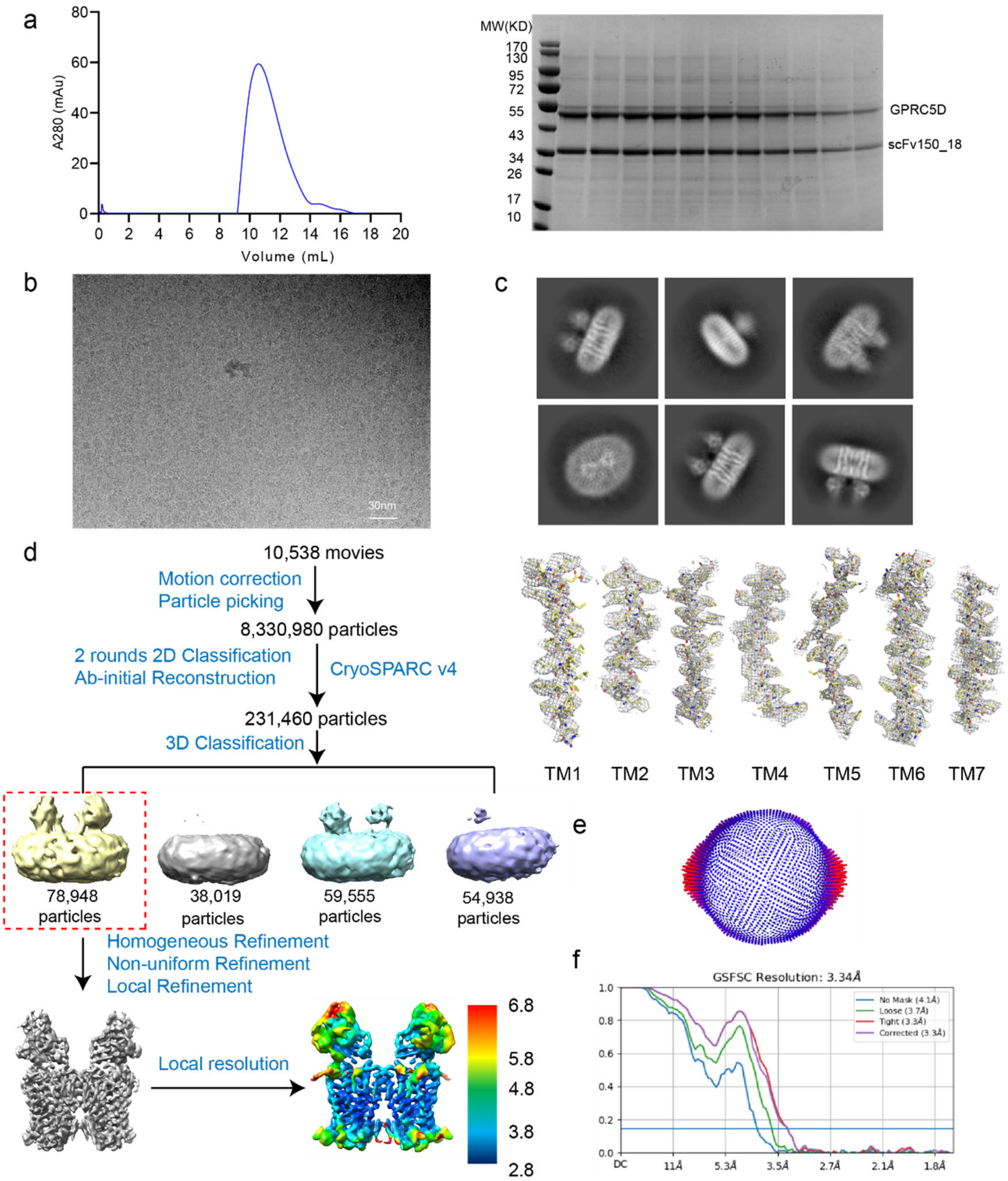

Supplementary Fig 3 | Workflow of cryo-EM sample preparation and data processing for GPRC5D-scFv150_18 complex. a Left panel, analytical size-exclusion chromatography (SEC) of the GPRC5D-scFv150_18 complex, and, right panel, SDS–PAGE analysis of the complex sample after SEC. **b, c** Representative cryo-EM micrograph and reference-free two-dimensional class averages of the GPRC5D-scFv150_18 complex. **d** Left panel, workflow of cryo-EM data processing for GPRC5D-scFv150_18 complex. The final map is colored according to the local resolution, and, right panel, EM maps for 7TMs of GPRC5D in this structure. **e** Angular distribution of the particles used for the final reconstruction of the GPRC5D-

scFv150_18 complex. **f** Gold-standard FSC curve, showing the overall nominal resolution at 3.34 Å.

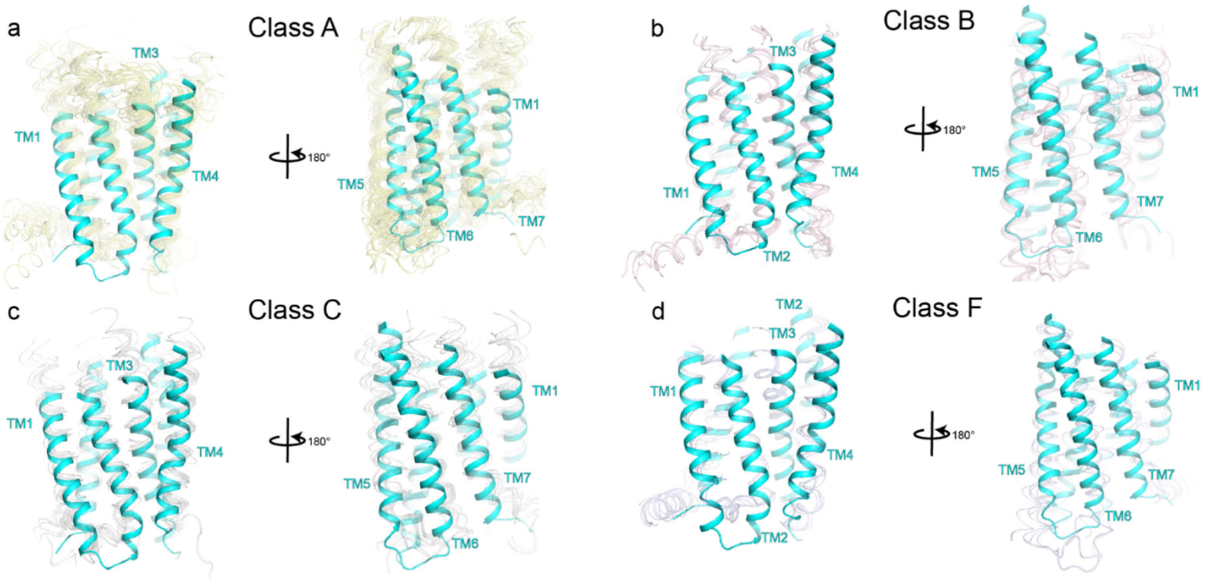

Supplementary Fig 4 | Structural comparison of 7TM domain between GPRC5D and representative GPCRs from classes A, B, C and F. a-d 7TM domain comparison of GPRC5D with Class A, Class B, Class C and Class F GPCRs.

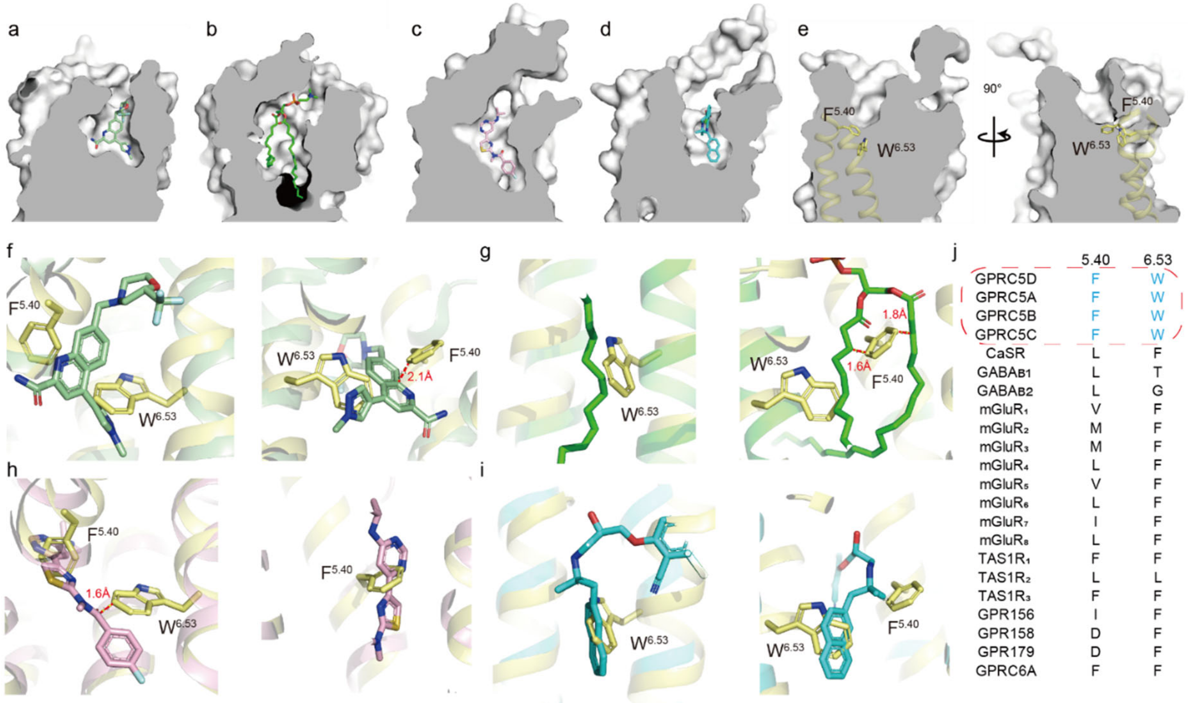

Supplementary Fig 5 | Pocket analysis of GPRC5D. a-d The transmembrane pockets of mGluR$_2$ (PDB: 7EPE), GABA$_B$ (PDB: 6WIV), mGluR$_1$ (PDB: 4OR2) and CaSR (PDB: 7M3J) with the receptor surface shown in gray and the ligand within the pocket represented in light green (**a**), green (**b**), pink (**c**) and cyan (**d**), respectively. **e** The corresponding pocket of GPRC5D, with the receptor surface shown in gray and the TM5-TM6 helices in yellow. **f-i** F$^{5.40}$ and W$^{6.53}$ of GPRC5D show steric clash with ligands of other Class C structures (light green: mGluR$_2$ (PDB: 7EPE); green: GABA$_B$ (PDB: 6WIV); pink: mGluR$_1$ (PDB: 4OR2); cyan: CaSR (PDB: 7M3J)). **j** Sequence comparison of residues 5.40 and 6.53 within Class C GPCRs. The red box represents the GPRC5 subfamily.

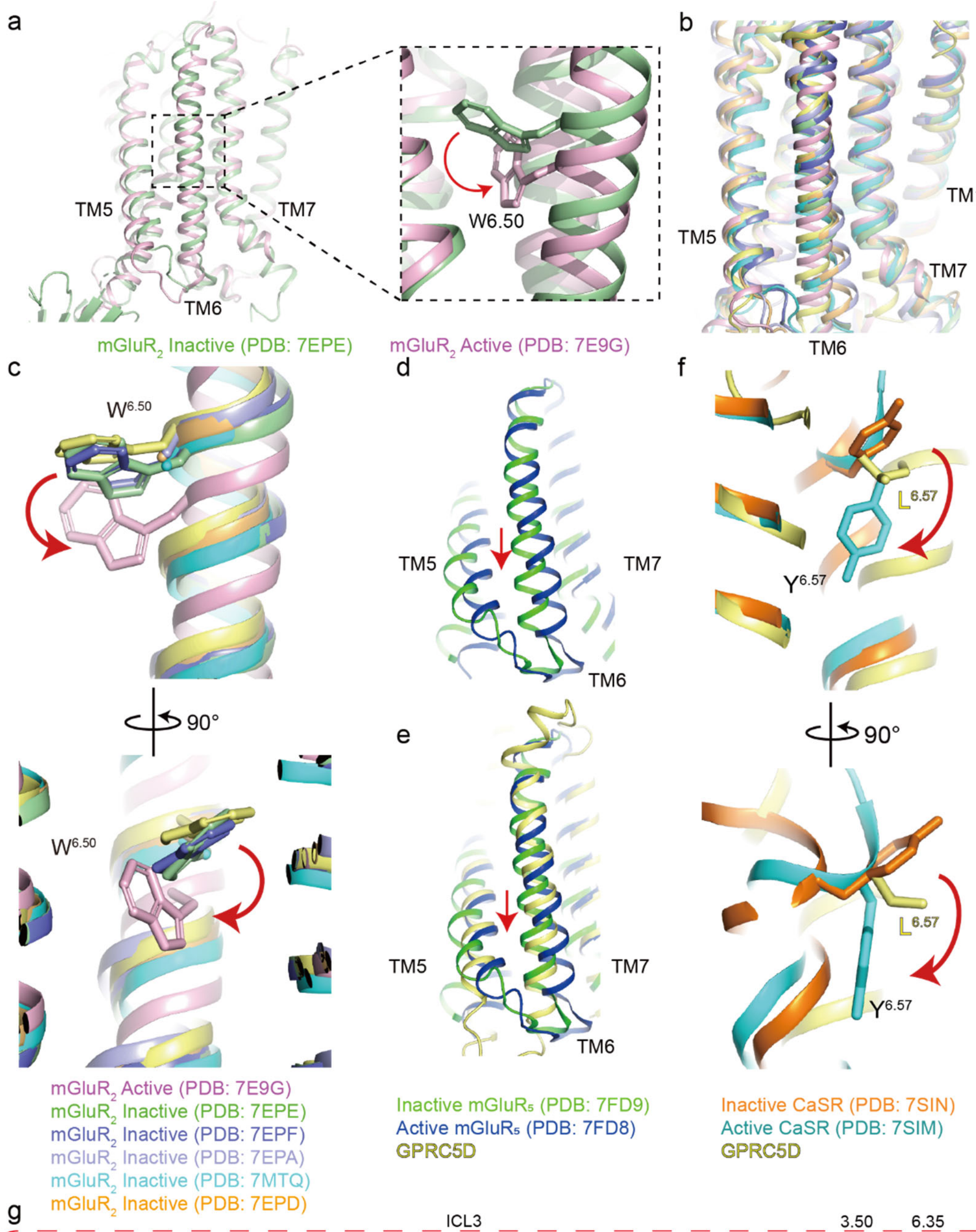

g

						ICL3					3.50	6.35	3.53
GPRC5D	F	C	G	P	C	E	N	W	K	Q	H	H	N
GPRC5A	F	C	G	S	F	T	G	W	K	R	H	H	S
GPRC5B	L	C	G	K	F	K	R	W	K	L	Q	N	R
GPRC5C	L	C	G	R	Y	K	R	W	R	K	H	H	A
CaSR	P	E	-	-	-	-	-	-	N	F	K	E	R
GABAB1	S	V	S	T	-	-	E	K	I	N	K	D	W
GABAB2	S	I	P	-	-	-	-	A	L	N	K	D	R
mGluR1	R	N	V	P	-	-	A	N	F	N	K	E	R
mGluR2	P	E	-	-	-	-	-	-	-	N	K	E	R
mGluR3	R	K	C	P	-	-	E	N	F	N	K	E	C
mGluR4	R	G	V	P	E	-	T	F	N	E	K	-	R
mGluR5	R	N	V	P	-	-	A	N	F	N	K	E	R
mGluR6	G	V	P	-	-	-	-	-	E	T	K	E	R
mGluR7	G	V	P	-	-	-	-	-	E	N	K	E	R
mGluR8	G	V	P	-	-	-	-	-	E	T	K	E	R
TAS1R1	P	E	-	-	-	-	-	-	N	Y	R	E	Q
TAS1R2	P	T	-	-	-	-	-	-	N	Y	R	E	Q
TAS1R3	P	G	-	-	-	-	-	-	C	Y	L	R	Q
GPR156	S	S	P	P	-	-	-	V	N	Q	K	S	R
GPR158	R	T	V	P	-	-	-	S	A	F	K	E	R
GPR179	R	A	V	L	-	-	-	S	A	F	K	E	R
GPRC6A	N	-	-	-	-	-	-	-	-	Y	K	E	K

Supplementary Fig 6 | Comparison of activation-related motifs/residues between

GPRC5D and other Class C receptors. a The swing of $W^{6.50}$ from the inactive (green) to active (pink) states of the $mGluR_2$. **b** TM6 comparison of GPRC5D with active (PDB: 7E9G) and inactive (PDB: 7EPE) $mGluR_2$. **c** Comparison of residue $W^{6.50}$ in the active and inactive states of $mGluR_2$ and GPRC5D. The active state of $mGluR_2$ is depicted in pink (PDB: 7E9G), while the inactive states are represented as green (PDB:7EPE), blue (PDB:7EPF), grey (PDB:7EPA), cyan (PDB:7MTQ), and orange (PDB:7EPD) in various reported structures. It is noteworthy that the conformation of $W^{6.50}$ in these inactive-state $mGluR_2$ structures are highly consistent and align well with $W^{6.50}$ in GPRC5D. **d-e** The comparison of TM6 between inactive (PDB: 7FD9) and active states of $mGluR_5$ (PDB: 7FD8) and GPRC5D. **f** The comparison of TM6 between inactive (PDB: 7SIN) and active (PDB: 7SIM) states of CaSR and GPRC5D. **g** **"**Ionic lock" sequence alignment in Class C GPCRs. The red box represents the GPRC5 subfamily, while the blue box indicates the E754 residue in ICL3 of $mGluR_2$ involved in forming the ionic lock. The blue residues denote conserved positively charged amino acids at positions 3.50 and 3.53, while the red indicates conserved negatively charged amino acids at position 6.35.

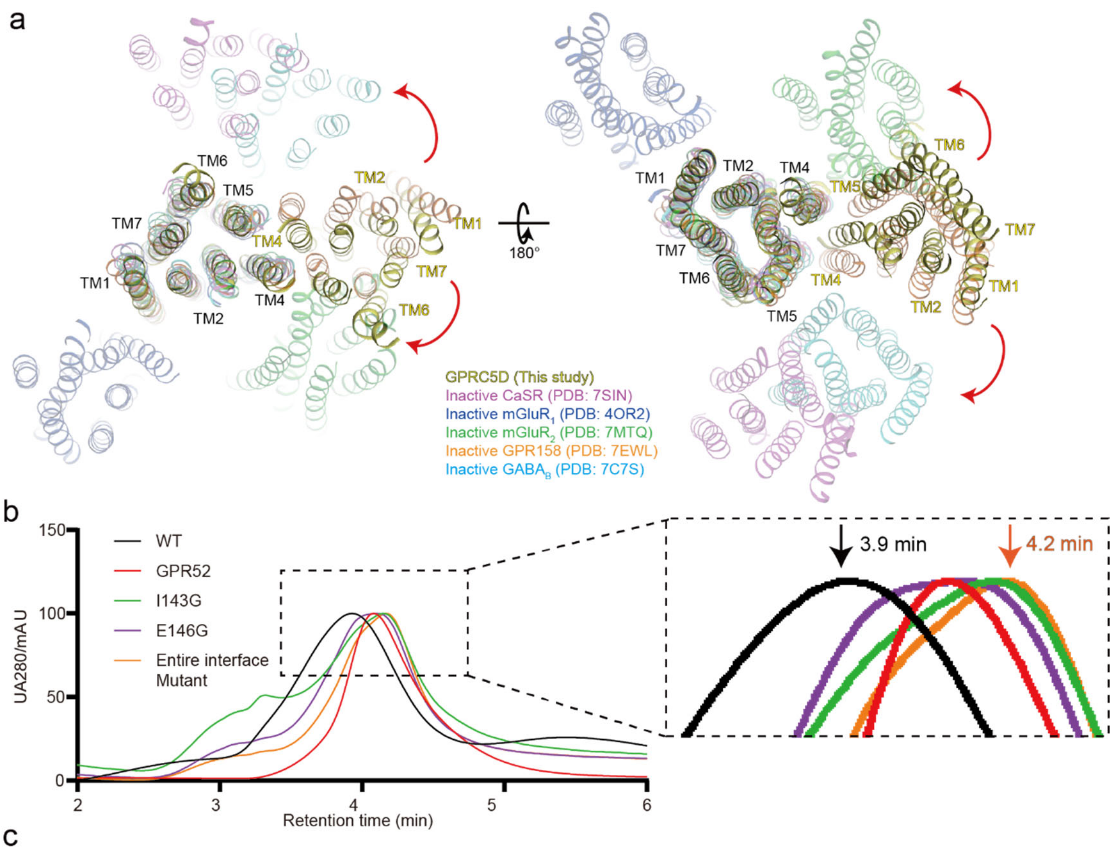

c

	4.45	4.46	--	4.49	--	4.53	4.54	--	4.61	--	5.35
GPRC5D	I	I	--	E	--	L	I	--	R	--	Q
GPRC5A	V	I	--	E	--	L	T	--	R	--	-
GPRC5B	I	I	--	E	--	L	T	--	R	--	A
GPRC5C	I	I	--	E	--	I	T	--	R	--	A
CaSR	V	I	--	I	--	T	A	--	S	--	H
GABAB1	L	T	--	I	--	V	D	--	H	--	S
GABAB2	C	I	--	C	--	V	D	--	R	--	E
mGluR1	T	L	--	T	--	M	E	--	M	--	N
mGluR2	L	I	--	A	--	V	E	--	G	--	N
mGluR3	V	M	--	V	--	L	E	--	G	--	N
mGluR4	L	G	--	V	--	V	D	--	H	--	D
mGluR5	G	I	--	A	--	M	E	--	D	--	N
mGluR6	V	G	--	A	--	A	R	--	H	--	D
mGluR7	L	G	--	I	--	V	D	--	N	--	D
mGluR8	L	G	--	V	--	V	D	--	H	--	D
TAS1R1	L	I	--	T	--	V	W	--	L	--	T
TAS1R2	V	I	--	I	--	A	T	--	S	--	N
TAS1R3	A	L	--	W	--	A	F	--	E	--	R
GPR156	I	L	--	T	--	T	D	--	Q	--	A
GPR158	W	F	--	G	--	S	V	--	Q	--	L
GPR179	G	F	--	V	--	G	A	--	H	--	H
GPRC6A	V	I	--	L	--	F	A	--	T	--	E

Supplementary Fig 7 | The comparison of GPRC5D dimer interface with other inactive-state Class C receptors. a The comparison between the dimer interface of GPRC5D and other Class C GPCRs. In order to illustrate the sharp differences of the dimer arrangement and interface between GPRC5D and canonical Class C GPCRs, we aligned one subunit of the dimers in representative Class C GPCRs with an anchoring subunit of GPRC5D to observe the divergent orientation of the other subunit. GPRC5D is depicted in yellow, GPR158 in orange (PDB: 7EWL), CaSR in pink (PDB: 7SIN),

$mGluR_1$ in blue (PDB: 4OR2), $mGluR_2$ in green (PDB: 7MTQ), and $GABA_B$ in cyan (PDB: 7C7S). The left side displays an extracellular view, while the right side represents an intracellular view. **b** The comparison of SEC retention time among different dimer interface mutants. Black curve represents the WT GPRC5D, which exhibits dimer retention time. GPR52 serves as the monomeric control. The entire interface mutant denotes a construct where all dimer interfaces have been mutated to glycine. **c** The sequence alignment of the GPRC5D dimer interface among Class C GPCRs. The red box represents the GPRC5 subfamily. The V/IxxE motif present within the GPRC5 subfamily is highlighted in red.

Supplementary Table 1 | Cryo-EM data collection, refinement and validation statistics

	GPRC5D-scFv150_18
**Data collection and processing**	
Magnification	105,000
Voltage (kV)	300
Electron exposure (e-/ Å^2)	60
Defocus range (μm)	-1.0 to -2.2
Pixel Size (Å)	0.832
Symmetry imposed	C2
Initial particle images (no.)	8,330,980
Final particle images (no.)	78,948
Map resolution (Å)	3.34
FSC threshold	0.143
Map resolution range (Å)	2.8 ~ 6.8
**Refinement**	
Map sharpening B factor (Å^2)	-113.2
Model composition	
Non-hydrogen atoms	6,827
Protein residues	986
B factors (Å^2)	
Protein	93.99
R.m.s. deviations	
Bond lengths (Å)	0.006
Bond angles (°)	0.887
Validation	
MolProbity score	1.98
Clash score	9.89
Ramachandran plot	
Favored (%)	92.59
Allowed (%)	7.41
Disallowed (%)	0.00

Supplementary Table 2 | Analysis of structural similarity on the transmembrane regions between GPRC5D and representative GPCRs from Classes A, C, and D. In the case of dimer structures, only one monomer is used for RMSD calculation.

Receptor	Family	PDB	RMSD (Å)
A2A	Class A	2YDV	13.281
CB1	Class A	5TGZ	14.267
CB2	Class A	5ZTY	16.186
D2R	Class A	6CM4	18.221
D3R	Class A	3PBL	10.243
D4R	Class A	5WIU	17.447
β2AR	Class A	2RH1	9.010
CCR2	Class A	5T1A	7.792
CCR5	Class A	4MBS	8.097
CCR7	Class A	6QZH	13.748
CCR9	Class A	5LWE	5.880
CXCR2	Class A	6LFL	10.337
CXCR4	Class A	3ODU	11.509
5HT1B	Class A	5V54	18.859
5HT2A	Class A	6WH4	11.403
5HT5A	Class A	7UM4	15.501
C5aR1	Class A	5O9H	17.317
APJ	Class A	7W0L	17.059
GPR52	Class A	6LI1	17.018
GPR20	Class A	8HS2	13.666
DOR	Class A	4N6H	12.946
KOR	Class A	4DJH	17.326
CaSR	Class C	7SIN	2.345
$GABA_{B1}$	Class C	7C7S	4.316
$GABA_{B2}$	Class C	7C7S	4.278
$mGluR_1$	Class C	4OR2	2.713
$mGluR_2$	Class C	7MTQ	1.964
$mGluR_3$	Class C	7WI8	5.282
$mGluR_4$	Class C	7E9H	8.189
$mGluR_5$	Class C	6N51	3.935
$mGluR_7$	Class C	7EPC	2.405
GPR158	Class C	7EWL	14.411

Ste2R	Class D	7AD3	17.439

Supplementary Table 3 | The dimer interface area of representative Class C GPCR homodimers at the transmembrane regions

Receptors	PDB	Area (Å^2)	State
GPRC5D	\	621.684	apo
GPR158	7EWL	804.956	apo
mGluR$_1$	4OR2	298.971	Inactive
CaSR	7SIN	48.018	Inactive
mGluR$_2$	7MTQ	104.754	Inactive
GABA$_B$	7C7S	383.415	Inactive

Note: The calculation of the dimer interface only focuses on the transmembrane helical region and does not include the VFT domain. During the calculation process, all lipids, small molecules, and fusion proteins were removed. The software used for the calculation is PyMOL 2.5.4.
