# Supplementary material for: The binding mechanism of an anti-multiple myeloma antibody to the human GPRC5D homodimer
Source: Nat Commun. 2024 Jun 19;15:5255. doi: 10.1038/s41467-024-49625-y (PMC11187071; doi:10.1038/s41467-024-49625-y)
Supplement: Supplementary file 3 — Reporting Summary [file 41467_2024_49625_MOESM3_ESM.pdf]

nature portfolio

Corresponding author(s): Fei Xu

Last updated by author(s): May 24, 2024

Reporting Summary

Nature Portfolio wishes to improve the reproducibility of the work that we publish. This form provides structure for consistency and transparency in reporting. For further information on Nature Portfolio policies, see our Editorial Policies and the Editorial Policy Checklist.

Statistics

For all statistical analyses, confirm that the following items are present in the figure legend, table legend, main text, or Methods section.

n/a	Confirmed	
☐	☒	The exact sample size (*n*) for each experimental group/condition, given as a discrete number and unit of measurement
☐	☒	A statement on whether measurements were taken from distinct samples or whether the same sample was measured repeatedly
☒	☐	The statistical test(s) used AND whether they are one- or two-sided<br>*Only common tests should be described solely by name; describe more complex techniques in the Methods section.*
☒	☐	A description of all covariates tested
☒	☐	A description of any assumptions or corrections, such as tests of normality and adjustment for multiple comparisons
☐	☒	A full description of the statistical parameters including central tendency (e.g. means) or other basic estimates (e.g. regression coefficient) AND variation (e.g. standard deviation) or associated estimates of uncertainty (e.g. confidence intervals)
☒	☐	For null hypothesis testing, the test statistic (e.g. *F*, *t*, *r*) with confidence intervals, effect sizes, degrees of freedom and *P* value noted<br>*Give P values as exact values whenever suitable.*
☒	☐	For Bayesian analysis, information on the choice of priors and Markov chain Monte Carlo settings
☒	☐	For hierarchical and complex designs, identification of the appropriate level for tests and full reporting of outcomes
☒	☐	Estimates of effect sizes (e.g. Cohen's *d*, Pearson's *r*), indicating how they were calculated

Our web collection on statistics for biologists contains articles on many of the points above.

Software and code

Policy information about availability of computer code

Data collection	Automated data collections on the Titan Krios were performed using Serial EM.
Data analysis	cryoSPARC 2.15,Phenix1.14, Coot 0.8.9, PyMOL 1.4.1, UCSF Chimera 1.15, Graph Pad Prism 8.0.

For manuscripts utilizing custom algorithms or software that are central to the research but not yet described in published literature, software must be made available to editors and reviewers. We strongly encourage code deposition in a community repository (e.g. GitHub). See the Nature Portfolio guidelines for submitting code & software for further information.

Data

Policy information about availability of data

All manuscripts must include a data availability statement. This statement should provide the following information, where applicable:

- Accession codes, unique identifiers, or web links for publicly available datasets
- A description of any restrictions on data availability
- For clinical datasets or third party data, please ensure that the statement adheres to our policy

Data supporting the findings of this manuscript are available from the corresponding authors upon reasonable request. The coordinate and EM map have been deposited in the Protein Data Bank, and the validation report is provided.

Research involving human participants, their data, or biological material

Policy information about studies with human participants or human data. See also policy information about sex, gender (identity/presentation), and sexual orientation and race, ethnicity and racism.

Reporting on sex and gender	N/A
Reporting on race, ethnicity, or other socially relevant groupings	N/A
Population characteristics	N/A
Recruitment	N/A
Ethics oversight	N/A

Note that full information on the approval of the study protocol must also be provided in the manuscript.

Field-specific reporting

Please select the one below that is the best fit for your research. If you are not sure, read the appropriate sections before making your selection.

☒ Life sciences ☐ Behavioural & social sciences ☐ Ecological, evolutionary & environmental sciences

For a reference copy of the document with all sections, see nature.com/documents/nr-reporting-summary-flat.pdf

Life sciences study design

All studies must disclose on these points even when the disclosure is negative.

Sample size	For cryo-EM studies, sample size was determined by availability of microscope. For the functional assays, no statistical methods were used to predetermine sample size. The sufficient sample size were chosen for statistical analysis.
Data exclusions	No data were excluded from the analysis
Replication	All the functional assay were conducted at least three independent experiments with technical repeats. All attempts at replication were successful.
Randomization	Randomization is not relevant to this study, since no experimental group was assigned in all experiments
Blinding	Blinding between receptor and antibody was measured by Fluorescence activated Cell Sorting (FACS).

Reporting for specific materials, systems and methods

We require information from authors about some types of materials, experimental systems and methods used in many studies. Here, indicate whether each material, system or method listed is relevant to your study. If you are not sure if a list item applies to your research, read the appropriate section before selecting a response.

Materials & experimental systems

n/a	Involved in the study	
☐	☒	Antibodies
☐	☒	Eukaryotic cell lines
☒	☐	Palaeontology and archaeology
☒	☐	Animals and other organisms
☒	☐	Clinical data
☒	☐	Dual use research of concern
☒	☐	Plants

Methods

n/a	Involved in the study	
☒	☐	ChIP-seq
☐	☒	Flow cytometry
☒	☐	MRI-based neuroimaging

Antibodies

Antibodies used	Mouse M2-fluorescein isothiocyanate (FITC) anti-FLAG antibody: Sigma, Cat#F4049, 1:100 diluted in TBS supplemented with 4% BSA and 20% viability staining solution 7-AAD (Invitrogen); goat anti-Human IgG (Fc specific)-FITC (fluorescein isothiocyanate) antibody:

	Sigma, Cat#F9512),1:100 diluted in TBS supplemented with 4% BSA; IgG150_18 was purified in-house.
Validation	The Mouse M2-fluorescein isothiocyanate (FITC) anti-FLAG antibody has been commercially obtained and validated by the validation data are avaliable from the respective manufacturer's website (https://www.sigmaaldrich.cn/CN/zh/product/sigma/f404); goat anti-Human IgG (Fc specific)-FITC (fluorescein isothiocyanate) antibody has been commercially obtained and validated by the validation data are avaliable from the respective manufacturer's website (https://www.sigmaaldrich.cn/product/sigma/f9512); IgG150_18 was validated by SDS-PAGE.

Eukaryotic cell lines

Policy information about cell lines and Sex and Gender in Research

Cell line source(s)	Spodoptera frugiperda Sf9 (Invitrogen, 12659017) ;HEK293T cells/HTLA cells (ATCC,CRL-11268).
Authentication	Authentication was done by the manufacturers by standard STR profiling analysis.
Mycoplasma contamination	All cell lines tested are negative for mycoplasma contamination.
Commonly misidentified lines (See ICLAC register)	No commonly misidentified cell lines were used.

Plants

Seed stocks	N/A
Novel plant genotypes	N/A
Authentication	N/A

Flow Cytometry

Plots

Confirm that:

- ☐ The axis labels state the marker and fluorochrome used (e.g. CD4-FITC).
- ☐ The axis scales are clearly visible. Include numbers along axes only for bottom left plot of group (a 'group' is an analysis of identical markers).
- ☐ All plots are contour plots with outliers or pseudocolor plots.
- ☐ A numerical value for number of cells or percentage (with statistics) is provided.

Methodology

Sample preparation	GPRC5D expressed HEK293T cells were incubated with IgG150_18, and then incubated with anti-Human IgG (Fc specific)-FITC.
Instrument	Guava easyCyte Instrument.
Software	Guava EasyCyte HT system.
Cell population abundance	2000 cells were used.
Gating strategy	Unexpressed HEK293T cells were used as negative control.

☐ Tick this box to confirm that a figure exemplifying the gating strategy is provided in the Supplementary Information.
